# Regulator of Angiogenesis and Vascular Function: A 2019 Update of the Vasoinhibin Nomenclature

**DOI:** 10.3389/fendo.2019.00214

**Published:** 2019-04-10

**Authors:** Jakob Triebel, Juan Pablo Robles, Magdalena Zamora, Gonzalo Martínez de la Escalera, Thomas Bertsch, Carmen Clapp

**Affiliations:** ^1^Institute for Clinical Chemistry, Laboratory Medicine and Transfusion Medicine, General Hospital Nuremberg and Paracelsus Medical University Nuremberg, Nuremberg, Germany; ^2^Instituto de Neurobiología, Universidad Nacional Autónoma de México, Querétaro, Mexico

**Keywords:** prolactin, growth hormone, placental lactogen, prolactin/vasoinhibin axis, 16K PRL

Proteolytic cleavage of prolactin (PRL), the human anterior pituitary hormone fundamental for lactation can generate vasoinhibin, a peptide-hormone with endocrine, paracrine, and autocrine effects not shared with its precursor. Vasoinhibin effects include the regulation of blood vessel growth, permeability, and dilation (1, 2), and non-vascular effects such as stimulation of vasopressin release (3), thrombolytic actions (4), inhibition of neurite outgrowth (5), and the stimulation of anxiety- and depression-related behaviors (6). Vasoinhibin signals through a still-unidentified receptor on endothelial cells distinct from the PRL-receptor and interacts with multiple binding partners (4, 7, 8). The role of vasoinhibin in biology and disease is evolving and its understanding requires the revision of its nomenclature, which is the purpose of this commentary (9).

The regulation of vasoinhibin generation occurs at the hypothalamo, the pituitary, and the target tissue levels and this organizational principle is described as the prolactin/vasoinhibin axis (10). A dysregulation of this axis is relevant in several diseases. Recent studies have focused on retinal disorders (11, 12), joint diseases (13), and pregnancy associated syndromes, for example diabetic retinopathy (11, 14), rheumatoid arthritis (13), peripartum-cardiomyopathy (15), and pre-eclampsia (16, 17). Two clinical trials in which vasoinhibin levels are the target of pharmacological interventions were initiated, one for the treatment of diabetic retinopathy and diabetic macular edema, and another for the treatment of peripartum cardiomyopathy (18, 19). The principles and rationales behind these clinical trials were recently reviewed (20). Landmark studies on the physiological and pathophysiological effects of vasoinhibin are presented in Table 1.

**Table 1:** Landmark original research articles and reviews highlighting physiology and pathophysiological effects of vasoinhibin.

**Brief description**	**Year**	**References**
**ORIGINAL RESEARCH ARTICLES**		
Model of the three-dimensional structure of vasoinhibin, and localization of its functional domain	2018	(21)
Clinical trial protocol on diabetic retinopathy and diabetic macular edema, pharmacological intervention into regulation of PRL/vasoinhibin axis	2018	(19)
Suppression of neurotrophic VEGF and NGF-induced effects	2017	(5)
Findings of a clinical trial on peripartum cardiomyopathy, pharmacological intervention into PRL/vasoinhibin axis	2017	(18)
Binding partners and profibrinolytic action	2014	(4)
Role on mammary gland involution in mice	2014	(22)
Effects on anxiety- and depression-like behaviors in rats	2014	(6)
Vasoinhibin gene therapy against diabetic retinopathy protects against VEGF- and diabetes-induced retinal vasopermeability in rats	2011	(14)
Hyperprolactinemia in rodents leads to vasoinhibin accumulation in the retina	2010	(11)
Cathepsin D generates vasoinhibin in rat anterior pituitary PRL secretory granules	2009	(23)
Inhibition of vasopermeability in diabetic retinopathy	2008	(24)
Impairment of cardiac capillary proliferation and function in peripartum cardiomyopathy	2007	(15)
Vasoinhibin gene therapy against tumor growth and metastasis	2007	(25)
Effect on endothelial cell dysfunction and low birth weight in preeclampsia	2007	(17)
Bone morphogenetic protein 1 generates vasoinhibin	2007	(26)
Matrix metalloproteases generate vasoinhibin	2006	(27)
Inhibition of angiogenesis and vasodilation in the rat retina by endogenous vasoinhibin	2005	(28)
Stimulation of vasopressin release	2003	(3)
Stimulation of ocular vascular regression in retinopathy of prematurity by endogenous vasoinhibin	2004	(29)
Inhibition of retinal angiogenesis in oxygen-induced retinopathy in mice	2004	(30)
Inhibition of tumor growth in human colon cancer cells transplanted into mice	2001	(31)
Proinflammatory effects in pulmonary fibroblasts and alveolar type II cells	2000	(32)
Vasoinhibin contains the N-terminal region of PRL	1999	(33)
Opposite effects of PRL and vasoinhibin on angiogenesis	1999	(34)
Inhibition of corneal angiogenesis by exogenous and endogenous vasoinhibin	1999	(35)
Cathepsin D generates vasoinhibin	1993	(36)
Inhibition of *in vitro* and *in vivo* angiogenesis	1993	(37)
Specific vasoinhibin binding sites in endothelial cell membranes	1992	(7)
Discovery of antiangiogenic properties	1991	(38)
Detection of vasoinhibin in the human pituitary gland and plasma	1985	(39)
Cleavage of PRL by target tissues	1983	(40)
Discovery of vasoinhibin as a functional PRL fragment in rat pituitary tissue	1980	(41, 42)
**REVIEW ARTICLES**		
Translational research, focus on diabetic retinopathy and peripartum cardiomyopathy	2017	(20)
Involvement of the PRL/vasoinhibin axis in rheumatoid arthritis	2016	(13)
First description of the PRL/vasoinhibin endocrine axis	2015	(10)
Pathophysiological role of vasoinhibin in peripartum cardiomyopathy	2014	(43)
Physiological and pathophysiological roles	2009	(2)
Actions on mammary gland	2008	(44)
Biology of vasoinhibin, vascular effects, and signal transduction	2006	(45)
Prolactin-, growth hormone-, and placental lactogen derived vasoinhibin and its effect on angiogenesis	2002	(46)

*Nearly 40 years of research since the discovery of vasoinhibin in 1980 resulted in a series of insights into the function and regulation of vasoinhibin. The latest developments feature clinical studies in diabetic retinopathy and peripartum cardiomyopathy, the first-ever in which vasoinhibin regulation is pharmacologically targeted. Also, the first three-dimensional model of vasoinhibin including a localization of its functional domain was communicated recently. The list of original research articles in this table comprise those considered landmarks by the authors, however, there are more relevant articles discussed and cited in the reviews listed at the end of the table*.

Historically, vasoinhibin was named “16 kDa PRL” or “16K fragment of prolactin” referring to the molecular mass of one of its isoforms and to PRL as its precursor (37, 38). With the introduction of a new nomenclature in 2006, the term was updated and changed to “vasoinhibin” (27, 45) (Figure 1). The introduction of the vasoinhibin nomenclature was triggered by the recognition that PRL fragments with inhibitory effects on blood vessels are not a single 16 kDa species, but rather a family of proteins with different molecular masses (10). As their functional and structural features are unique and contrast with those of full-length PRL, it was recognized that these proteins are sole hormones, and should not bear the same designation as PRL. Further, it was discovered that fragments of growth hormone (GH) and placental lactogen (PL), hormones closely related to PRL, demonstrate similar antiangiogenic properties (34, 46). As a family, they were collectively named “vasoinhibins,” inspired by their principal effects, the inhibition of blood vessel growth, and control of blood vessel function (45).

**Figure 1:**
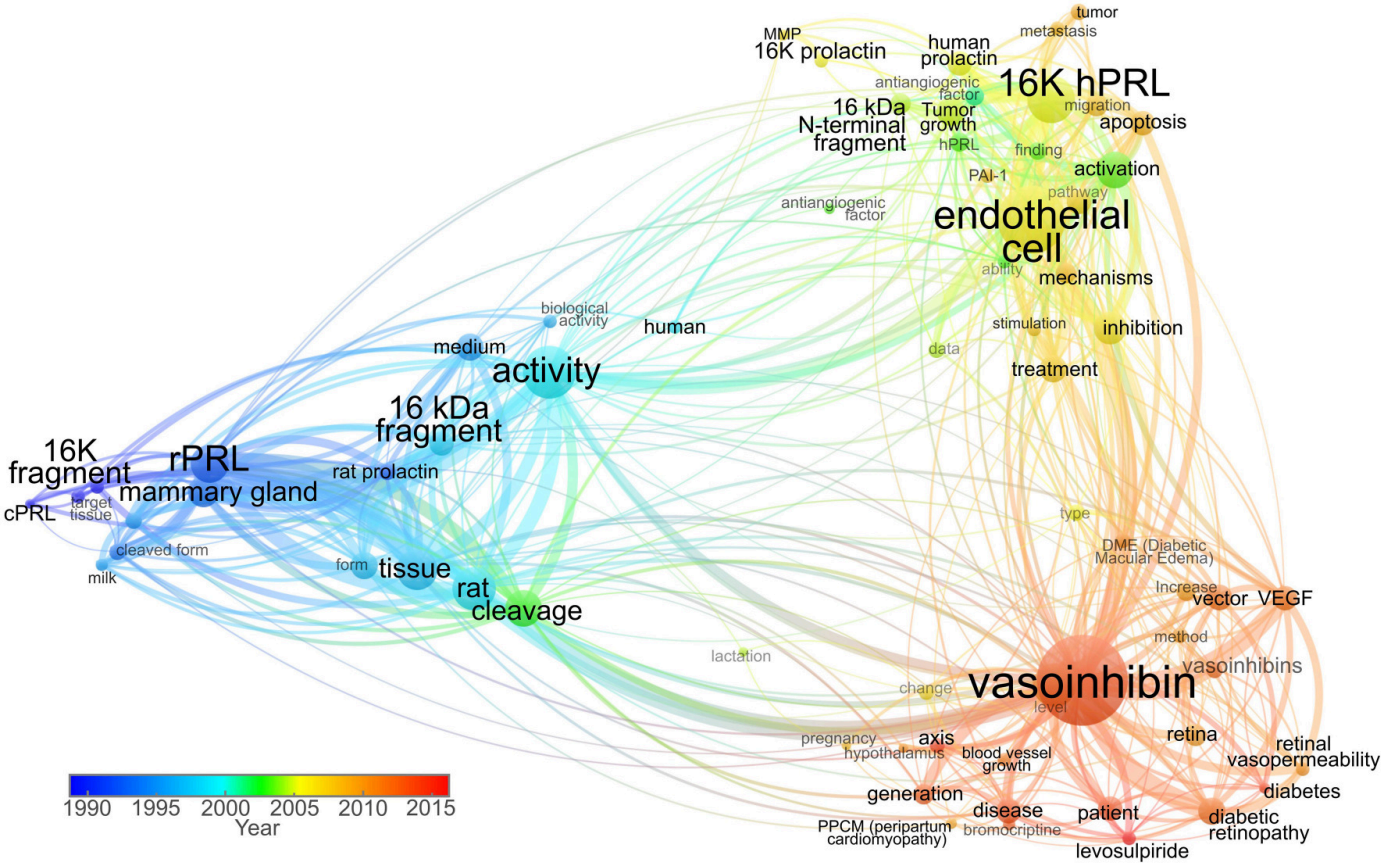
Term co-occurrence map analyzing the use of vasoinhibin-related names in titles and abstracts throughout time. Scientific articles published between 1980 and 1999 used terms such as “16K fragment,” “cPRL” (cleaved prolactin), and “16 kDa fragment” as valid nomenclature. The predominant “16K hPRL” term was then used in combination with “16K prolactin” and “16 kDa N-terminal fragment” up to 2006, when the “vasoinhibin” term was introduced. In recent years, the “vasoinhibin” word shows higher frequency, whereas the 16K-related shows progressive lower incidence. The size of the node represents the frequency of term appearance. The lines between terms indicate relations with smaller distances representing stronger associations. The color indicates average year of term appearance. The map was created and visualized using VOSviewer software tool (47) in which the network was constructed employing 71 terms with more than 60% relevance, selected from 119 words surpassing the seven-appearance threshold, in titles and abstracts of 93 research publications accessed through Scopus.

In the years ensuing the introduction of the vasoinhibin nomenclature, a heterogeneous use of the terminology was observed (Figure 1). Some kept using the historic designation “16 kDa PRL fragment,” in some instances due to the convenience of highlighting the PRL-related context of its action (48); others used “16 kDa vasoinhibin,” the term in its plural form “vasoinhibins,” or a combination of these designations (9, 15, 22). In an attempt to identify the precursor of vasoinhibin, and to discriminate it from vasoinhibin of other origin (for example GH or PL-derived), it was also referred to as “prolactin-derived vasoinhibins” or “prolactin-related vasoinhibin” (6, 49). Also, numbering the vasoinhibin isoforms in order to designate their origin and to state the molecular mass of each isoform was suggested (50). Of note, in communication with journals and in peer-review procedures, the designation “vasoinhibin” was confused with “vasohibin,” a new term for a protein unrelated to vasoinhibin which was introduced around the same time than the vasoinhibin nomenclature (51).

It became clear, that the heterogeneous use of other terms for vasoinhibin is unfavorable as it complicates orientation and introduces inaccuracies when using search engines. Moreover, a uniform nomenclature to correctly and completely annotate the growing biological and clinical information about vasoinhibin in data bases is required. The use of the terms “16 kDa PRL” and the like, as listed above, can no longer be recommended, as they are outdated (Figure 1) and do not conform with the International Protein Nomenclature Guidelines (IPNG) (52) in which both, the use of the molecular mass (16 kDa), and ambiguity (PRL) is discouraged. Therefore, in line with the published literature, and with the IPNG, we suggest using the term “vasoinhibin” for a peptide hormone fulfilling the following criteria:

- The protein is generated by post-translational processing, i.e., proteolytic cleavage, of PRL, GH, or PL (UniProt ID P01236, P01241, and P0DML2).
- The protein demonstrates inhibition of endothelial cell proliferation and inhibition of angiogenesis in *in vitro* and *in vivo* bioassays, respectively.
- Having the vasoinhibin bioactive domain architecture and solution structure is emerging as a third criterion (21), but requires further experimental validation and is, therefore, projected as a future criterion.

The criteria A, B, and C correspond to the IPNG rank of sources, whereas criteria B and C also correspond to experimental reports and domain architecture, respectively. Other rank denominations (established and maintained database authorities), models (Hidden Markov models), and signatures are not yet available.

The criteria are based on present knowledge and should not be interpreted as final as it is possible that vasoinhibin of other origin than PRL, GH, and PL will be discovered. In case of similar biological activity and domain architecture, such protein would consequently receive the designation vasoinhibin or vasoinhibin-domain containing protein. Therefore, we also recommend maintaining the inclusive designation of “vasoinhibin-family” when addressing the entirety of known and/or undiscovered vasoinhibin (-like) hormones. Also, as vasoinhibin is a pleiotropic hormone, it remains to be investigated whether all its diverse effects are mediated by one or more bioactive domains.

The present commentary reflects on the history of the nomenclature used for vasoinhibin (Figure 1), and recommends, based on the latest literature and the protein nomenclature guidelines, how to handle this nomenclature in scientific publications and data bank entries. We believe that consideration of the present recommendations will improve the accuracy of scientific communication and hereby benefit the field.

## Author Contributions

JT, JPR, and CC wrote the manuscript. MZ, GMdelaE, and TB edited and revised the manuscript. All authors approved the manuscript.

### Conflict of Interest Statement

The authors declare that the research was conducted in the absence of any commercial or financial relationships that could be construed as a potential conflict of interest.
